# Macrophagic HDAC3 inhibition ameliorates Dextran Sulfate Sodium induced inflammatory bowel disease through GBP5-NLRP3 pathway

**DOI:** 10.7150/ijms.94592

**Published:** 2024-05-19

**Authors:** Na Che, Yang Zhang, Shu Zhang, Xiangxi Kong, Ying Zhang, Shukun Wang, Zengqiang Yuan, Yajin Liao

**Affiliations:** 1 Department of neurology, The Second Affiliated Hospital, Hengyang Medical School, University of South China, Hengyang, China.; 2 Center on Translational Neuroscience, College of Life & Environmental Science, Minzu University of China, Beijing, China.; 3 School of Basic Medical Sciences, Anhui Medical University, Hefei, China.; 4 The Brain Science Center, Beijing Institute of Basic Medical Sciences, No. 27 Taiping Road, Haidian District, Beijing, China.; 5 Jiangsu Province Key Laboratory of Anesthesiology, Jiangsu Province Key Laboratory of Anesthesia and Analgesia Application Technology, Xuzhou Medical University, Xuzhou, China.

**Keywords:** HDAC3, GBP5, NLRP3, macrophage, inflammatory bowel disease

## Abstract

Inflammatory bowel disease (IBD) is a chronic inflammatory intestinal disease, characterized by dysregulated immune response. HDAC3 is reported to be an epigenetic brake in inflammation, playing critical roles in macrophages. However, its role in IBD is unclear. In our study, we found HDAC3 was upregulated in CX3CR1-positive cells in the mucosa from IBD mice. Conditional knockout (cKO) of Hdac3 in CX3CR1 positive cells attenuated the disease severity of Dextran Sulfate Sodium (DSS)-induced colitis. In addition, inhibition of HDAC3 with RGFP966 could also alleviate the DSS-induced tissue injury and inflammation in IBD. The RNA sequencing results revealed that Hdac3 cKO restrained DSS-induced upregulation of genes in the pathways of cytokine-cytokine receptor interaction, complement and coagulation cascades, chemokine signaling, and extracellular matrix receptor interaction. We also identified that Guanylate-Binding Protein 5 (GBP5) was transcriptionally regulated by HDAC3 in monocytes by RNA sequencing. Inhibition of HDAC3 resulted in decreased transcriptional activity of interferon-gamma-induced expression of GBP5 in CX3CR1-positive cells, such as macrophages and microglia. Overexpression of HDAC3 upregulated the transcriptional activity of GBP5 reporter. Lastly, conditional knockout of Hdac3 in macrophages (Hdac3 mKO) attenuated the disease severity of DSS-induced colitis. In conclusion, inhibition of HDAC3 in macrophages could ameliorate the disease severity and inflammatory response in colitis by regulating GBP5-NLRP3 axis, identifying a new therapeutic avenue for the treatment of colitis.

## Introduction

Inflammatory bowel disease (IBD) is a chronic, nonspecific, and inflammatory intestinal disease, which is defined as a dysregulated immune response to environmental triggers 1-3. The psychological symptoms, sleep quality, and quality of life of IBD patients are also disturbed. CX3C chemokine receptor 1 (CX3CR1) is the receptor for fractalkine, and is mainly expressed in monocytes, T-cell subsets, NK cells and dendritic cell 4. Inhibition of fractalkine-CX3CR1 axis is reported to be beneficial for IBD 5. Among the above cell types, CX3CR1 positive macrophage is one subset of the monocytes resident in the colonic mucosa 6-8. It is reported that the number of CX3CR1 positive macrophages is significantly increased during the acute phase of mouse and human colitis 9, 10. These CX3CR1 high-expressed macrophages recruit CX3CR1 low-expressed macrophages into local colon tissues with colitis by releasing C-C motif ligand 2 (CCL2) and CCL7 9, 11. The accumulated macrophages secrete a large amount of pro-inflammatory factors, such as interleukin-23a (IL-23a) and IL-1β 11. Blockage of pro-inflammatory factors in CX3CR1 positive macrophage or knockout of CX3CR1 could protect mice from disease severity and tissue injury in Dextran Sulfate Sodium (DSS) colitis, and decrease the recruitment of monocytes 7. However, the mechanism of CX3CR1-positive monocytes in colitis is unclear.

Histone deacetylase 3 (HDAC3) is regarded as an epigenomic brake in macrophage alternative activation 12. Knockout of HDAC3 in macrophages and microglia results in decreased expression of pro-inflammatory factors and increased expression of type 2 T helper cell cytokines 12, 13. Mechanically, type I interferon pathway and nuclear factor kappa-B (NF-κB) pathways are suppressed in HDAC3-deficient macrophage. Our previous study reveals that HDAC3 is also important for the expression of pro-inflammatory factors in microglia, another subtype of CX3CR1 positive monocytes 14. Conditional knockout of *Hdac3* in CX3CR1-positive monocytes (*Hdac3* cKO) restrains oxidative stress and self-DNA induced microglial activation 14. CX3CR1 positive macrophage are the resident monocytes and are significantly expanded in the acute phase inflammation in colitis, however, whether HDAC3 is involved in the process is unknown.

Interferon gamma (IFN-γ) is an early response cytokine during microbial infection, and is secreted to extracellular space to induce the expression of a large amount of pro-inflammatory genes by activating its receptor 15. Breakup of intestinal homeostasis and aberrant immune response to commensal microbiota are the major characterisations of colitis 16. Aberrant production of IFN-γ is also observed in the development of colitis, and inhibition of IFN-γ displays some benefits for colitis therapy 17. Guanylate-Binding Protein 5 (GBP5) is one of the IFN-γ stimulated genes, which can be hardly detected in cells without IFN-γ stimulation, however, it is robustly increased upon reinduction with IFN-γ 17-19. Recent studies reveal that GBP5 is upregulated in human and mouse colitis and promotes the development of IBD 20, 21. Knockout of GBP5 could significantly alleviate the tissue damage in DSS-induced colitis 20, suggesting GBP5 may be a critical target gene of IFN-γ that is involved in the development of colitis 19. What's more, GBP5 is identified as an activator of NOD-like receptor (NLR) family, pyrin domain-containing-3 (NLRP3) inflammasome. It's reported that GBP5 selectively promotes the assembly of NLRP3 inflammasomes in response to microbial and soluble dangerous agents 22-24. Activation of NLRP3 inflammasomes is associated with severe tissue damage in IBD 25, 26. The activation of NLRP3 inflammasomes promotes the secretion of mature IL-1β and induces pyroptosis by cleavage of gasdermin D (GSDMD) 27, 28. Besides inducing pyroptosis, GSDMD could also mediates the secretion of pro-IL-1β to promote intestinal inflammation in colitis 29.

Here, we found the expression of HDAC3 was upregulated in the macrophage of colitis model mice. Inhibition of HDAC3 and conditional knockout of *Hdac3* could protect mice from colon damage and inflammasome activation in colitis. Mechanically, the current study revealed that HDAC3 promotes the development of colitis in macrophages by positively regulating IFN-γ-induced expression of GBP5 and enhancing the activation of NLRP3 inflammasomes in GBP5-dependent manners.

## Materials and Methods

### Animal strains

HDAC3 floxp mice (Stock No: 024119 | Hdac3flox) were purchased from the Jackson Laboratory (Sacramento, CA, USA) and preserved in our laboratory 14. CX3CR1cre transgenic mice (Stock No: 021160) and Lyz2-Cre transgenic mice (Stock No: C001003) were purchased from the Cyagen Biotech. All mice were maintained in a specific pathogen-free animal facility (syxk-2019-003) and handled in accordance with the institute's guidelines for the care and use of laboratory animals.

### Induction of colitis

Apart from the control group, all other groups received 2.5% DSS diluted in drinking water for 7 days. Meanwhile, the control group received equal amounts of distilled water. For inhibition of HDAC3 *in vivo* with its specific antagonist, RGFP966 (#S7229, Selleck, China) was dissolved in DMSO to a concentration of 30 mg/mL and diluted in 30% (w/v) hydroxypropyl-β-cyclodextrin (#HK388-5g, Bio Basic Inc, Canada) and 100 mM sodium acetate (pH 5.4) to a final concentration of 3 mg/mL before injection. Then, a 30 mg/kg dose of RGFP966 was administered intraperitoneally twice per day for 7 consecutive days. Mice were executed on day 8, colon length was measured and opened longitudinally, and colonic tissues were collected for further study.

### Isolation and culture of primary macrophages

Primary peritoneal macrophages were isolated from adult wild-type and HDAC3 cKO mice using the method described previously 28. Briefly, the mice were sacrificed by the neck breaking method and immersed in 75% ethyl alcohol for 5 min. Macrophages were collected by washing the peritoneal cavity with ice-cold RPMI-1640 medium (#C11875500BT, Gibco, China). After centrifugation, the cell pellet was resuspended in fresh RPMI-1640 medium supplemented with 10% heat-inactivated FBS, 1% penicillin/streptomycin solution and seeded in culture plates at 5x105/ml. The non-adherent cells were removed 6 h post seeding and the adherent macrophages were further cultured for subsequent experiments. For inducing the expression of GBP5, recombinant mouse IFN-γ was added to the medium at 100 ng/ml 24 h post seeding plus 10 μM RGFP966 or equal volume of DMSO. For activation of NLRP3 inflammasome, cells were then exposed to 1 μg/ml lipopolysaccharide (LPS) in Opti-MEM medium plus 1% fetal bovine serum for 3.5 h at 6 h post adding IFN-γ and RGFP966, and followed by treating with 6.7 ng/ml nigericin (Nig) for 30 min. Lastly, the cell culture medium and the cells were both harvested for further analysis.

### Real-time quantitative polymerase chain reaction (RT-qPCR)

Total RNA was extracted from the colonic tissues using TRIzol reagent (#15596026, Invitrogen), and then cDNA was synthesized using a One-Step First-strand cDNA synthesis kit (#AT311-02, Transgen, Beijing, China). SYBR Green-based real-time qPCR (#A304, GenStar, Beijing, China) was performed to measure gene expression on Studio Q3 (ABI, USA). The gene expression levels were normalized with b-actin gene as the reference gene according to the 2-ΔΔCT method. The primers used here were as below. β-actin (forward): 5′-GGCTGTATTCCCCTCCATCG-3′, β-actin (reverse): 5′-CCAGTTGGTAACAATGCCATGT-3′, HDAC3 (forward): 5′-GCCAAGACCGTGGCGTATT-3, HDAC3 (reverse): 5′-GTCCAGCTCCATAGTGGAAGT-3, GBP5 (forward): 5′-CAGACCTATTTGAACGCCAAAGA-3′, GBP5 (reverse): 5′-TGCCTTGATTCTATCAGCCTCT-3′, PAK1 (forward): 5′-GAAACACCAGCACTATGATTGGA-3′, PAK1 (reverse): 5′-ATTCCCGTAAACTCCCCTGTG-3′.

### RNA sequencing for colonic tissues

For colonic tissues RNA sequencing, total RNA was extracted using TRIzol reagent and the genomic DNA was removed with RNAse-free DNAse I (#2270A, Takara, China), and the quality of the extracted RNA was determined using a NanoDrop 2000 spectrophotometer and Qubit 2.0 fluorometer. Then, the cDNA libraries were generated from the RNA using NEBNext® Ultra™ RNA Library Prep Kit for Illumina® (#E7770S, New England Biolabs, USA). The libraries were sequenced on an Illumina Hiseq 4000 platform, deep sequencing of mRNA profiles was performed by Annoroad Gene Technology Co., Ltd (Beijing, China); 150 bp paired-end reads were generated. 10G raw data for each sample were obtained and the clean data were analyzed by Annoroad Gene Technology. The fragments per kilobase million (FPKM) values of each gene from each library were used for Kyoto Encyclopedia of Genes and Genomes (KEGG) analysis and Gene-Set Enrichment Analysis (GSEA). GSEA is a computational method that determines whether an *a priori* defined set of genes shows statistically significant, concordant differences between two biological states.

### RNA sequencing for purified microglia

For primary CX3CR1-positive monocyte RNA sequencing, WT and HDAC3 cKO primary microglia were used in this study. Adult mouse brain was dissociated into single-sell suspension with Adult Brain Dissociation Kit (#130-107-677, Miltenyi Biotec, Germany) and the microglia were isolated for the mouse brain with anti-CD11b microbeads (#130-093-634, Miltenyi Biotec, Germany) plus MS columns (#130-122-727, Miltenyi Biotec, Germany) according to the manufacturer's instructions. Then, total RNA was extracted as above, and the cDNA libraries were generated from the RNA using a SMARTer® Universal Low Input RNA Kit for Sequencing (#634938, Takara). Then, the libraries were sequenced and the results were analyzed as mentioned above.

### Immunohistochemistry and stain

4% Polyformaldehyde-fixed paraffin-embedded intestine tissues from inflamed and non-inflamed sites of IBD mouse were sectioned at 5 μm in thickness and collected onto glass slides. Antigen retrieval was performed in 10 mM sodium citrate (pH6.0) at 95 ℃ for 15 min. GBP5 (#13220-1-AP, 1:100, Proteintech, China) was stained with VECTASTAIN ABC Kit (Vector labratories, USA) according to the protocol of the manufacturer. The alcian blue-PAS Staining (#G1285, Solarbio, China) and Hematoxylin-Eosin staining (#G1120-3x100ml, Solarbio) were performed according to the manufacturer's instructions. The images were collected with Slide Scanning System Pannoramic MIDI (3DHISTECH, Hungary). Quantitation of the GBP5 signals was performed with ImageJ with IHC-Toolbox plugins.

### Immunofluorescence and imaging

The tissue section was prepared as in IHC method. Primary antibodies included MUC2 (#ab272692, 1:200, Abcam, UK) and GSDMD (#ab219800, 1:200, Abcam, UK). Secondary antibodies included Alexa Fluor 546 conjugated goat antirabbit antibodies (Invitrogen, United States). Cell nuclei were stained with 40,6-diamidine-20-phenylindole dihydrochloride (DAPI, Sigma-Aldrich, United States). Slides were visualized with a Ti-A1 confocal microscope (Nikan, Japan).

### Western blot

Cells or tissues were lysed with RIPA lysis buffer comprising a cocktail of protease (#B14001, Selleck) and phosphatase inhibitors (#4906845001, Roche, USA). Total protein concentrations were measured using the BCA assay (#P0010, Biyuntian, China), and the proteins were separated by SDS-PAGE and then transferred to a NC membrane (#10401396/0.22μm, Whatman, USA). The membrane was blocked with 5% nonfat milk in Tris-buffered saline and incubated with primary antibodies overnight at 4 °C, followed by horseradish peroxidase-conjugated secondary antibodies for protein detection. The primary antibodies used in the present study were anti-HDAC3 (#A2139, 1:1000, Abclonal, China), anti-NLRP3 (#AG-20B-0014, 1:1000, AdipoGen, USA), anti-Casp-1 (#Ag-20B-0042, 1:1000, AdipoGen, USA), anti-ASC (#67824, 1:1000, Cell Signaling Technology, USA), anti-IL-1β (#AF-401-NA; 1:1000, R&D Systems, USA), GBP5(#13220-1-AP, 1:1000), and anti-β-actin (#P01L082, 1:1000, Gene-Protein Link, China).

### Cloning and plasmid construction

To construct the GBP5 reporter plasmid, gDNA was extracted from BV2 cells using the EasyPure® Genomic DNA kit (#EE101-01, TransGen Biotech, China) according to the manufacturer's instructions for mammalian cells. The promoter region of GBP5 (from approximately 2000 bp upstream of the ATG codon of GBP5) was amplified by PCR with PrimeSTAR GXL DNA polymerase (#R050A, Takara) and the primers as below. Forward primer: 5′-GGGGTACCGGCAAGTGCAGTCTAAAGCG-3′, reverse primer: 5′-CCCAAGCTTGTCTCTTAAGAAAAACTGAAG-3′. The PCR product was purified with gel extraction kit (#28704, QIAGEN, USA) according to the manufacturer's instructions. It was then digested with KpnI (#R3142S, New England Biolabs) and HindIII (#R3104T, New England Biolabs) at 37 °C for 2 h and purified with gel extraction kit. The purified fragment was then ligated with a KpnI and HindIII double-digested pGL3-basic vector and transformed into competent *E. coli* (DH5α) cells. To identify cells containing the plasmid of interest, the cells were cultured in Luria-Bertani medium with ampicillin and subjected to Sanger sequencing. The Flag-HDAC3 overexpression plasmid was preserved in our laboratory previously. All plasmids for transfection were purified using the QIAGEN Plasmid Maxi kit (#12163, QIAGEN).

### Dual-luciferase assay

The GBP5 reporter plasmid (50 ng/mL) and TK-Renilla plasmid (10 ng/mL) (used as a reference reporter here) were co-transfected into HEK293T cells together with the Flag-HDAC3 plasmid or Flag-vector plasmid. The cells were then treated with HDAC inhibitors (the concentration of RGFP966 and RGFP109 were both used at 10 μM) or vehicle 6 h post-transfection. The cells were harvested 24 h post-transfection, and the luciferase activity was determined using the Dual-Luciferase Assay System (#E1910, Promega, USA) and microplate reader (Spark 10M, Tecan, Switzerland).

### Statistical analysis

All data are expressed as the mean ± standard error of the mean of three independent experiments. The significance of differences was assessed by unpaired Student's t test (two groups) or one-way analysis of variance followed by Tukey's multiple comparisons test (three or more groups) with GraphPad Prism 8.0 software. Differences were considered significant at P < 0.05.

## Results

### HDAC3 is upregulated in CX3CR1-positive monocytes in DSS-induced colitis

**Figure 1:**
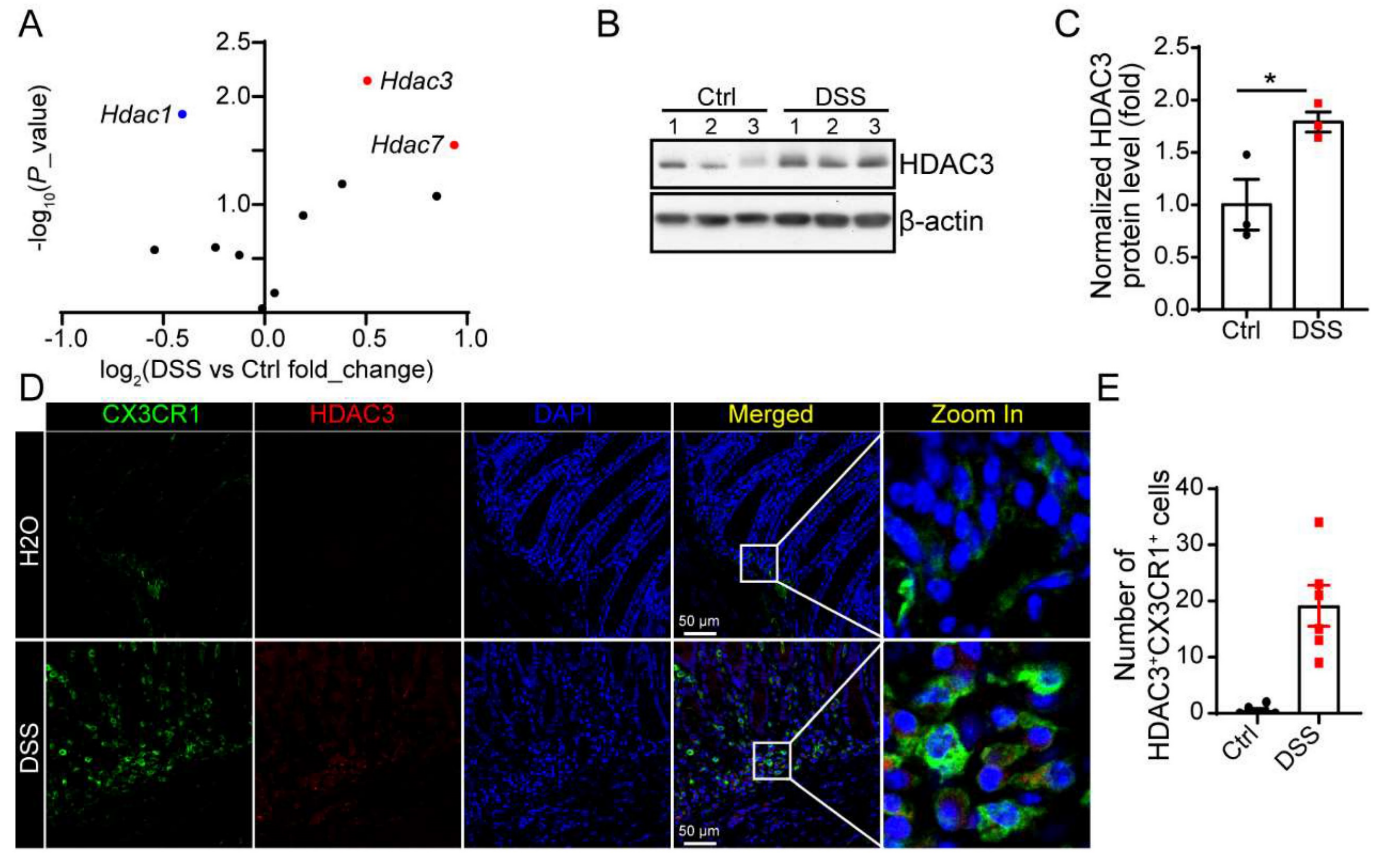
** HDAC3 is upregulated in CX3CR1-positive monocytes in DSS-induced colitis. (A)** The scatter diagram showed changes in the expression of the genes of HDAC family in the colon mucosa from DSS-induced colitis model mice. (**B** and **C**) The protein levels of HDAC3 and β-actin in colon mucosa from mice administered with water (n=3) or Dextran Sulfate Sodium (DSS)/water solution (n=3) were determined by western blot and the gray values were analyzed by Image J. (**D** and **E**) The protein levels of HDAC3 and CX3CR1 in colon mucosa from mice administered with water (n=6) or DSS/water solution (n=6) were determined by immunostaining and the pictures were capture by con-focal microscope. Annotations: * indicates p < 0.05, ** indicates p < 0.01 by Student's t-test (for 2 groups comparation).

Previous study proves that intestinal epithelial cell (IEC) specific deletion of HDAC3 results in increased susceptibility to intestinal damage and inflammation. However, in our study, we found in DSS-induced colitis, the mRNA levels and protein levels of HDAC3 were increased significantly (Figure 1A-C). The immunostaining results showed that HDAC3 was majorly co-localized with CX3CR1 in the mucosa from the DSS group, the marker gene for monocyte (Figure 1D and E).

### Conditional knockout of *Hdac3* in CX3CR1-positive monocytes attenuated DSS-induced colitis

**Figure 2:**
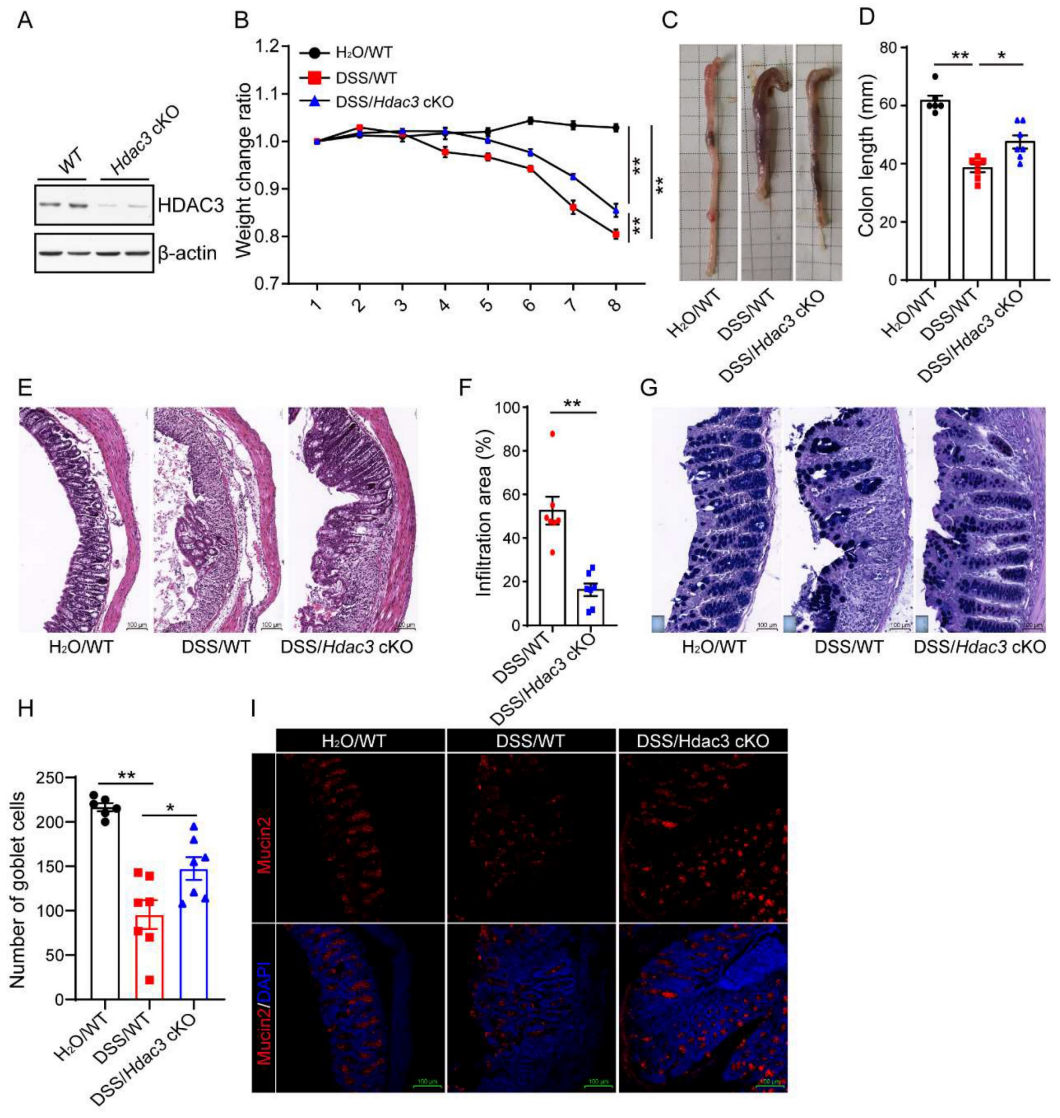
** Conditional knockout of *Hdac3* in CX3CR1-positive monocytes attenuated DSS-induced colitis.** (**A**) Primary microglia were isolated from the adult mouse brain of wild type (WT) and *Hdac3* cKO mice, and the protein levels of HDAC3 were determined by western blot. (**B**) The bodyweight change ratio of WT (n=7) and *Hdac3* cKO (n=7) mice administered with 2.5% DSS/water solution or water solely (n=6). (**C** and **D**) The length of colon from WT (n=7) and *Hdac3* cKO (n=7) mice administered with 2.5% DSS were determined and analyzed. (**E** and **F**) Typical H&E staining of colon tissue sections from WT (n=7) and *Hdac3* cKO (n=7) mice administered with 2.5% DSS/water solution and the percentage of the inflammatory cell infiltration and structurally damaged area were analyzed. (**G** and **H**) Typical Alcian Blue Periodic Acid Schiff (AB-PAS) staining of colon tissue sections from WT (n=7) and *Hdac3* cKO (n=7) mice administered with 2.5% DSS/water solution or water solely (n=6). (**G**) The number of goblet cells were counted (**H**). (**I** and **J**) The goblet cells were labeled with anti-Mucin2 antibody (**I**) and the mucin2 positive cells were counted (**J**). Annotations: * indicates p < 0.05, ** indicates p < 0.01 by ANOVA (for 3 groups comparation) or Student's t-test (for 2 groups comparation).

To verify whether CX3CR1-positive monocytes HDAC3 is involved in the development of colitis, we conditionally knocked out HDAC3 in CX3CR1 positive monocytes (HDAC3 cKO) by crossing HDAC3f/f mice with CX3CR1cre mice. The western blot result showed that the protein levels were significantly downregulated in HDAC3 cKO microglia, one type of CX3CR1 positive monocytes (Figure 2A). Then, the wild type (WT) and HDAC3 cKO mice were administered with 2.5% DSS for 8 days, and the bodyweight change ratio were recorded. The results displayed that the bodyweights of WT mice were significantly decreased from 4 days post administration of DSS, and conditional knockout of HDAC3 ameliorated the bodyweight decrease levels during DSS-induced colitis (Figure 2B). Then, we analyzed the colon length of those at 8th day post administration of DSS. The results showed that the colon lengths were significantly shorter than those from the water control group, and knockout of HDAC3 in CX3CR1 positive monocytes alleviated the colon length shortening induced by DSS (Figure 2C and D). In concordance with these results, conditional knockout of HDAC3 also effectively improved histopathological injuries, including the disruption of mucosal epithelium (Fig. 2E and F), infiltration of inflammatory cells (Figure 2E and F), and loss of goblet cells (Figure 2G-J).

### Inhibition of HDAC3 with RGFP966 ameliorated DSS-induced colitis

**Figure 3:**
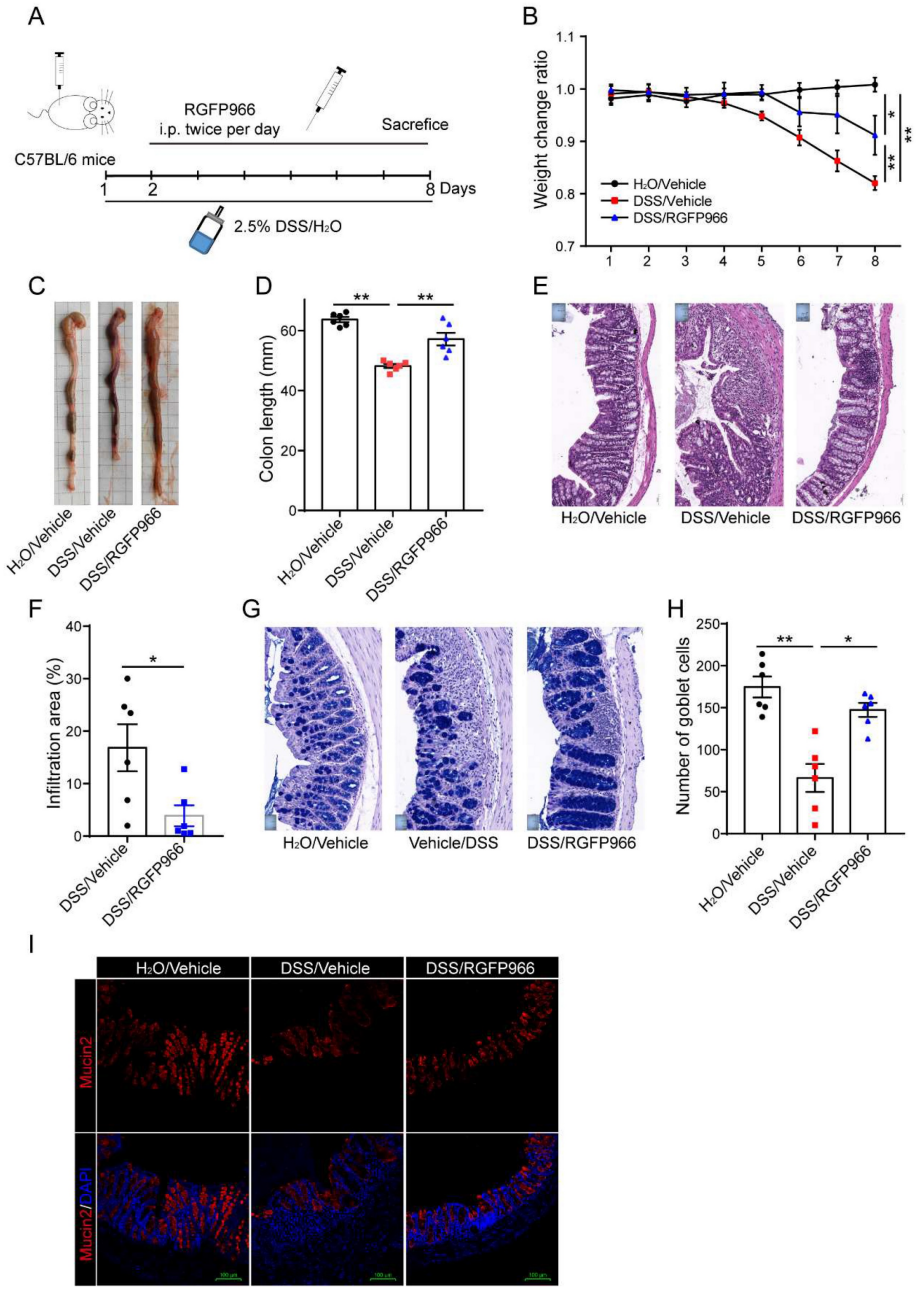
** Inhibition of HDAC3 with RGFP966 ameliorated DSS-induced colitis.** (**A**) Design of the experiments. (**B**) The bodyweight change ratio of WT mice administered with 2.5% DSS/water solution with or without administration of RGFP966 and from the mice administered with water solely (n=6 for each group). (**C** and **D**) The length of colon from WT mice administered with 2.5% DSS/water solution with or without administration of RGFP966 and from the mice administered with water solely were determined and analyzed (n=6 for each group). (**E** and **F**) Typical H&E staining of colon tissue sections from WT mice administered with 2.5% DSS/water solution with or without administration of RGFP966 and the percentage of the inflammatory cell infiltration and structurally damaged area were analyzed (n=6 for each group). (**G**-**I**) The goblet cells in the colon tissue from WT mice administered with 2.5% DSS/water solution with or without administration of RGFP966 and from the mice administered with water solely were stained with AB-PAS staining (**G** and **H**) and anti-Mucin2 antibody (n=6 for each group) (**I**). Annotations: * indicates p < 0.05, ** indicates p < 0.01 by ANOVA (for 3 groups comparation) or Student's t-test (for 2 groups comparation).

Further, we studied whether pharmaceutic inhibition of HDAC3 could ameliorate the disease severity as well. RGFP966, a HDAC3-specific inhibitor, was administered via i.p. injection twice per day from the 2nd day post administration of DSS (Figure 3A). The results displayed that DSS-induced bodyweight loss was alleviated by RGFP966 (Figure 3B). Consistent with this, DSS-induced colon length shortening was also rescued by administration of RGFP966 (Figure 3C and D). In addition, administration of RGFP966 via i.p. also effectively improved histopathological injuries, including the disruption of mucosal epithelium (Figure 3E and F), infiltration of inflammatory cells (Figure 3E and F), and loss of goblet cells (Figure 3G-I).

### *Hdac3* deficiency in CX3CR1-positive monocytes dampened the migration and inflammation of immune cells in DSS-induced colitis

**Figure 4:**
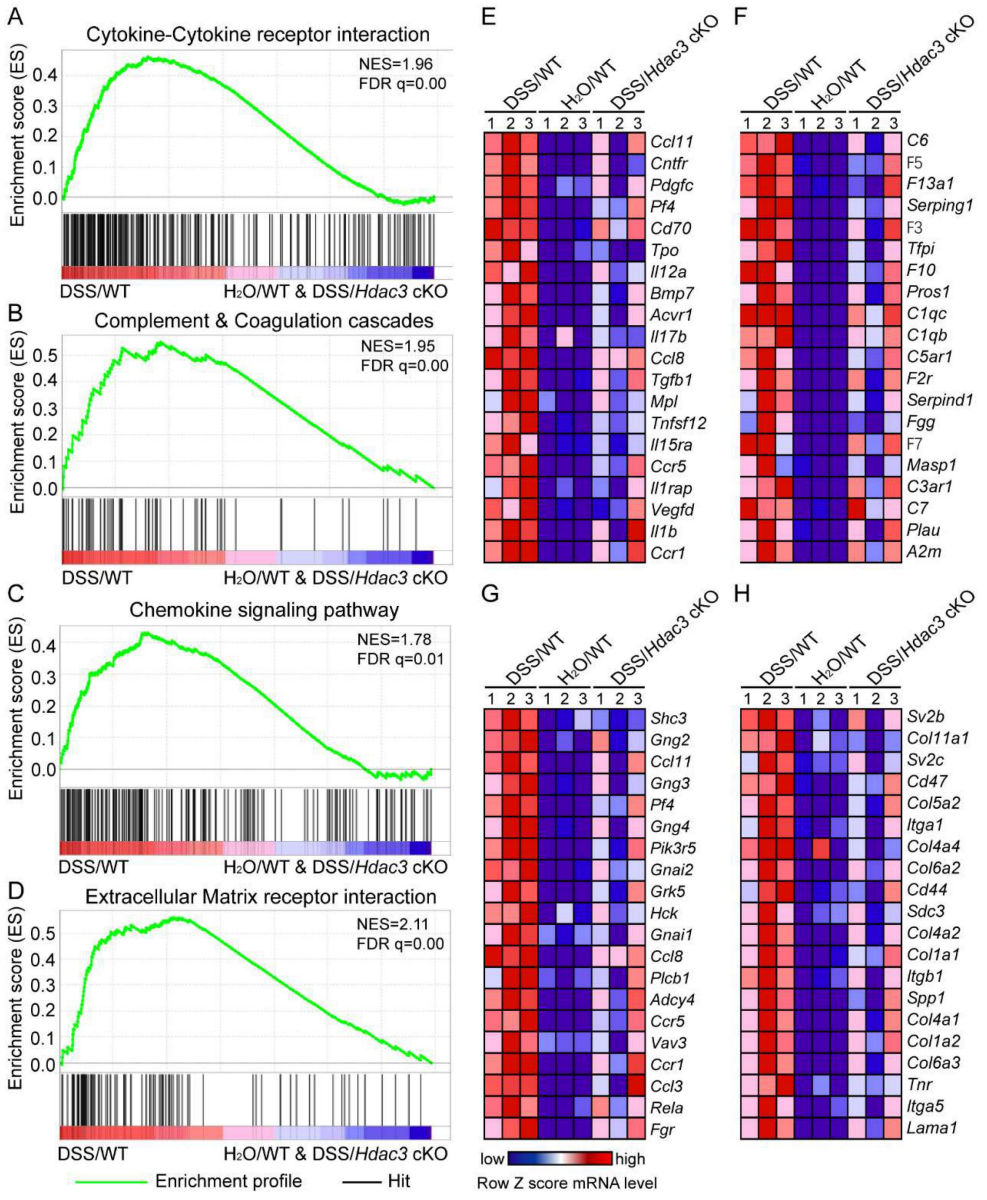
***Hdac3*-deficient in CX3CR1 positive monocytes dampened the migration and inflammation of immune cells in DSS-induced colitis.** (**A**-**D**) Gene set enrichment analysis (GSEA) showed the cytokine-cytokine receptor interaction (**A**), complement and coagulation cascades (**B**), chemokine signaling (**C**), extracellular matrix (ECM) receptor interaction (**D**) were all significantly enriched in the DSS/WT group. (**E**-**F**) Heatmap resulted from the GSEA displayed the top 20 upregulated genes in pathways of cytokine-cytokine receptor interaction (**E**), complement and coagulation cascades (**F**), chemokine signaling (**G**), ECM receptor interaction (**H**) in the colonic mucosa from DSS/WT group, water/WT group and DSS/*Hdac3* cKO group (n=3 for each group).

To clarify the mechanism of HDAC3 in CX3CR1-positive monocytes in DSS-induced colitis, we then performed RNA sequencing to analyze the gene expression profiles in the colonic mucosa. The RNA sequencing results were then analyzed by GSEA. The results indicated that the KEGG pathway including cytokine-cytokine receptor interaction (Figure 4A), complement and coagulation cascades (Figure 4B), chemokine signaling (Figure 4C), and extracellular matrix (ECM) receptor interaction (Figure 4D) were all significantly enriched in the DSS/WT group. Further analysis revealed that the top 20 leading genes which were upregulated in the above pathways from DSS/WT group, were downregulated by knockout of *Hdac3* in CX3CR1 positive monocytes (Figure 4E-H). These bioinformatic analysis results were in coordination with the above histopathological score.

### HDAC3 might upregulate the expression of GBP5 to potentiate NLRP3 inflammasome activation in CX3CR1-positive monocytes in colitis

**Figure 5:**
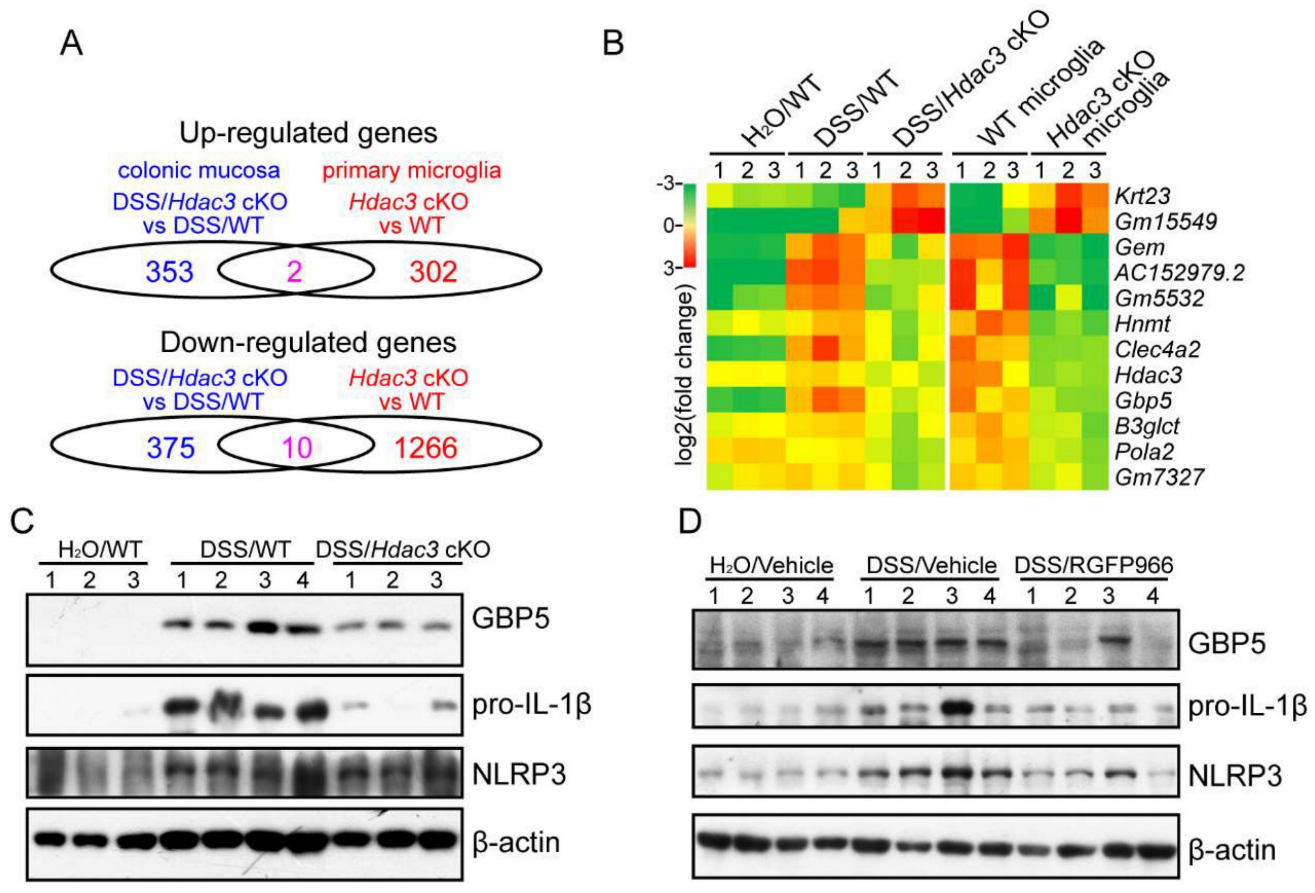
** HDAC3 might upregulate the expression of GBP5 to potentiate NLRP3 inflammasome activation in CX3CR1-positive monocytes in colitis.** (**A**) The number of significantly upregulated and downregulated genes in the colonic mucosa or primary microglia from *Hdac3* cKO mice identified by RNA sequencing (n=3 for each group). (**B**) Heatmap represents the mRNA levels of the shared genes in the colonic mucosa or primary microglia of each group. (**C**) The protein levels of GBP5, IL-1β, NLRP3 and β-actin in the colonic mucosa from WT (n=4) and *Hdac3* cKO (n=3) mice administered with 2.5% DSS/water solution or water solely (n=3) were detected by western blot. (**D**) The protein levels of GBP5, IL-1β, NLRP3 and β-actin in the colonic mucosa from WT mice administered with 2.5% DSS/water solution with or without administration of RGFP966 and from the WT mice administered with water solely were detected by western blot (n=4 for each group).

To find the target genes that regulated by HDAC3 in the development, we compared the RNA sequencing results from colonic mucosa and primary microglia. The results revealed that 2 genes were significantly upregulated and 10 genes were significantly downregulated in both the colonic mucosa and the monocytes from the Hdac3 cKO mice (Figure 5A and B). Among the genes, Gbp5 has been previously reported to be upregulated in colonic mucosa, and plays a pro-inflammatory role in the development of colitis by potentiating the activation of NLRP3 inflammasomes 20. In our study, we found GBP5 was upregulated in DSS/WT group, and was dampened in DSS/Hdac3 cKO group.

In addition, the mRNA levels were significantly downregulated in HDAC3 knockout CX3CR1-positive monocytes (Figure 5B). As result, we further analyzed the protein levels of GBP5 and NLRP3 inflammasome components in the colonic mucosa. The results indicated that the expression of GBP5 was upregulated in the DSS/WT group, and restrained in the DSS/Hdac3 cKO group (Figure 5C). The expression of IL-1β was decreased in the DSS/Hdac3 cKO group as well (Figure 5C). Consistent with this, RGFP966 treatment also alleviated DSS-induced upregulation of GBP5 and IL-1β (Figure 5D). Differently, the expression of NLRP3 was also downregulated by RGFP966 (Figure 5D).

### HDAC3 upregulates the IFN-γ induced transcription of GBP5 *in vitro*

**Figure 6:**
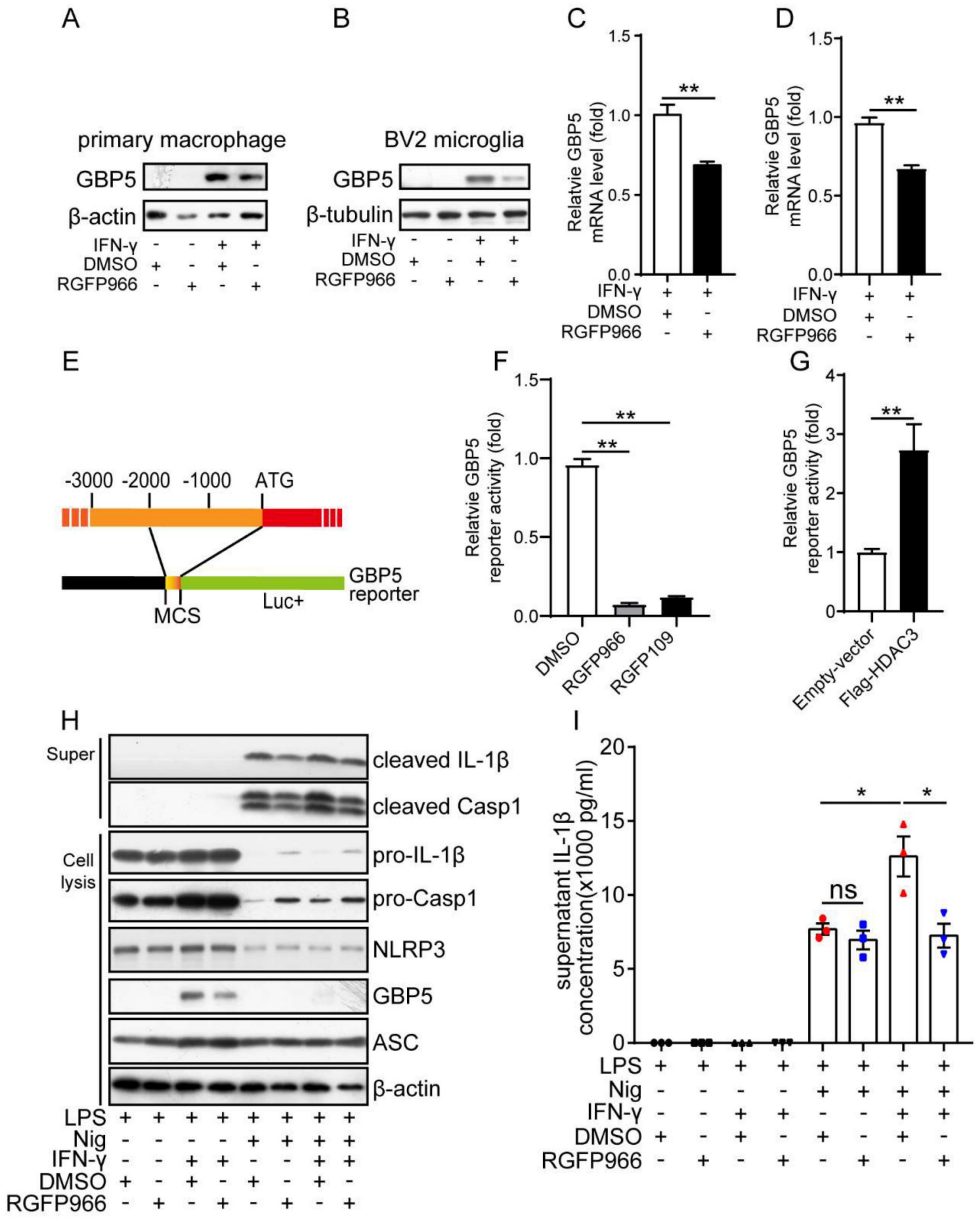
** HDAC3 potentiated the activation of NLRP3 inflammasomes by promoting IFN-γ induced expression of GBP5.** (**A** and **B**) The protein levels of GBP5 and β-actin or β-tubulin in primary macrophages (**A**) or BV2 microglia (**B**) treated with IFN-γ (100 ng/ml) for 6 h with or without RGFP966 (10 μM) pretreatment were detected by western blot. (**C** and **D**) The mRNA levels of GBP5 in primary macrophages (**C**) or BV2 microglia (**D**) treated with IFN-γ (100 ng/ml) for 6 h with or without RGFP966 (10 μM) pretreatment were detected by real-time quantitative PCR and normalized to the mRNA levels of β-actin (n=3). (**E**) Schematic diagram of the GBP5 reporter. (**F**) Cells were co-transfected with GBP5 reporter plasmid and TK-Renilla plasmids, treated with RGFP966 (10 μM), RGFP109 (10 μM) or vehicle, and harvested 24 h post-transfection for the dual-luciferase assay (n=3). (**G**) Plasmids encoding Flag-HDAC3 or empty vector were transfected into HEK293T cells with the GBP5 reporter and TK-Renilla reference plasmids, and the cells were harvested for the dual-luciferase assay 24 h post-transfection (n=3). (**H** and **I**) Primary microglia pretreated with or without RGFP966 (10 μM) for 30 min, were then exposed to IFN-γ (100 ng/ml) for 6 h, and followed by treated with 1 μg/ml LPS for 3.5 h, then stimulated with nigericin for another 30 min and the supernatant and cell were harvested for analysis the protein levels of cleaved IL-1β, cleaved casp1, pro-IL-1β, pro-Casp1, NLRP3, GBP5, ASC and β-actin by western blot (**H**) or enzyme-linked immunosorbent assay (n=3) (**I**). Annotations: the data represent at least two-independent experiments, and * indicates p < 0.05, ** indicates p < 0.01 by ANOVA (for 3 groups comparation) or Student's t-test (for 2 groups comparation).

As the above results suggest GBP5 may be a target gene of HDAC3, we then analyzed whether the expression of GBP5 was by HDAC3. The western blot results showed that GBP5 was only expressed in the macrophage under the stimulation of IFN-γ (Figure 6A). And its protein levels were downregulated by inhibition of HDAC3 with RGFP966 in both macrophages (Figure 6A) and microglia (Figure 6B). The RT-qPCR results further proved that the mRNA levels of GBP5 induced by IFN-γ were also downregulated in RGFP966-exposed macrophage (Figure 6C) and microglia (Figure 6D). These results suggest HDAC3 may transcriptionally regulate the expression of GBP5. Then, we constructed a Gbp5 promoter reporter by cloning the 2000 bp upstream of the initial codon of Gbp5 (Figure 6E), followed by analysis of the reporter gene activities under inhibition or overexpression of HDAC3 with dual luciferase reporter assay. The results indicated that the reporter gene activities were downregulated by RGFP966 and RGFP109 (another HDAC3 inhibitor) (Figure 6F), while its activities were upregulated in cells overexpressed with Flag-HDAC3 (Figure 6G). These results further confirmed that HDAC3 enhanced the transcription of GBP5 under the stimulation of IFN-γ. Then, we tested whether RGFP966 affected the IFN-γ potentiated activation of NLRP3 inflammasome. The results showed that IFN-γ pre-treatment increased the expression of cleaved IL-1β and cleaved Casp1, while RGFP966 abolished the potentiation effects of IFN-γ (Figure 6H). Consistently, the expression of secreted IL-1β was also increased by IFN-γ, which was impaired by RGFP966 as well (Figure 6I).

### Conditional knockout of *Hdac3* in CX3CR1-positive monocytes attenuated DSS-induced colitis

**Figure 7:**
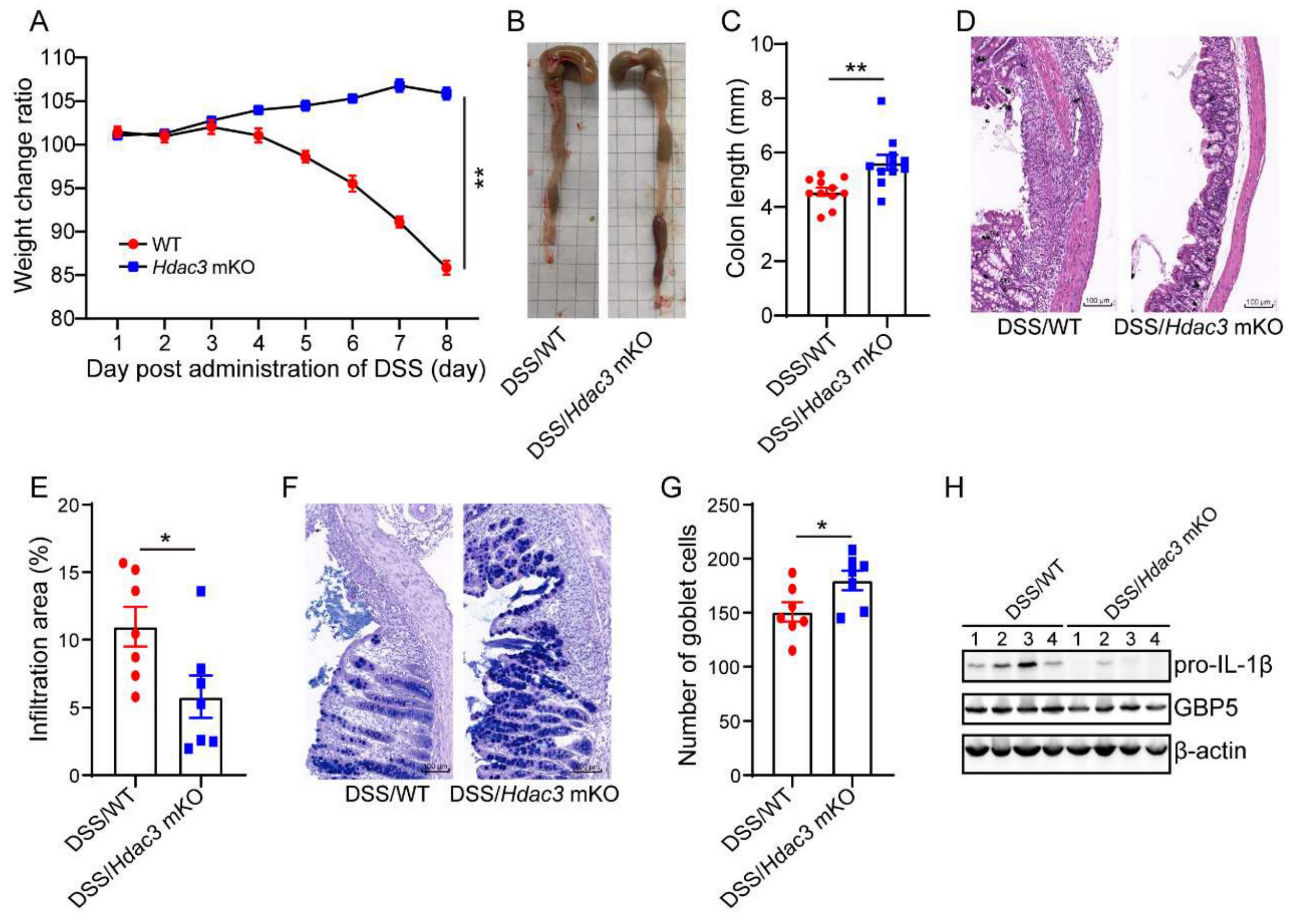
** Conditional knockout of *Hdac3* in macrophages attenuated DSS-induced colitis.** (**A**) The bodyweight change ratio of WT (n=10) and *Hdac3* mKO (n=10) mice administered with 2.5% DSS/water solution. (**B** and **C**) The length of colon from WT (n=7) and *Hdac3* mKO (n=7) mice administered with 2.5% DSS were determined and analyzed. (**D** and **E**) Typical H&E staining of colon tissue sections from WT (n=7) and *Hdac3* mKO (n=7) mice administered with 2.5% DSS/water solution and the percentage of the inflammatory cell infiltrated and structure damaged area were analyzed. (**F** and **G**) Typical AB-PAS staining of colon tissue sections from WT (n=7) and *Hdac3* mKO (n=7) mice administered with 2.5% DSS/water solution. (**H**) The expression of pro-IL-1β, GBP5 and β-actin in the colon from WT (n=7) and *Hdac3* mKO (n=7) mice administered with 2.5% DSS/water solution were determined by western blot. Annotations: * indicates p < 0.05, ** indicates p < 0.01 by Student's t-test (for 2 groups comparation).

As the above results suggest that HDAC3 upregulates IFN-γ-induced expression of GBP5, we then asked whether specific deletion of HDAC3 in macrophages could protect mice from DSS-induced colitis. We performed conditional knockout of HDAC3 in macrophages (HDAC3 mKO) by crossing HDAC3f/f mice with Lyz-IRES-iCRE mice. Then, the mice were administered with 2.5% DSS for 8 days, and the bodyweight change ratios were recorded. The results displayed that the bodyweight of WT mice was significantly decreased at day 5, while no decreased was observed in the HDAC3 mKO group (Figure 7A). Then, we analyzed the colon length and the results showed that the colon length of WT mice was shorter than that of HDAC3 mKO mice (Figure 7B and C). Consistently, HDAC3 mKO also effectively improved histopathological injuries, including the disruption of mucosal epithelium (Figure 7D and E), infiltration of inflammatory cells (Figure 7D and E), and loss of goblet cells (Figure 7F and G). In addition, the expression of IL-1β and GBP5 were downregulated in HDAC3 mKO group (Figure 7H).

## Discussion

**Figure 8:**
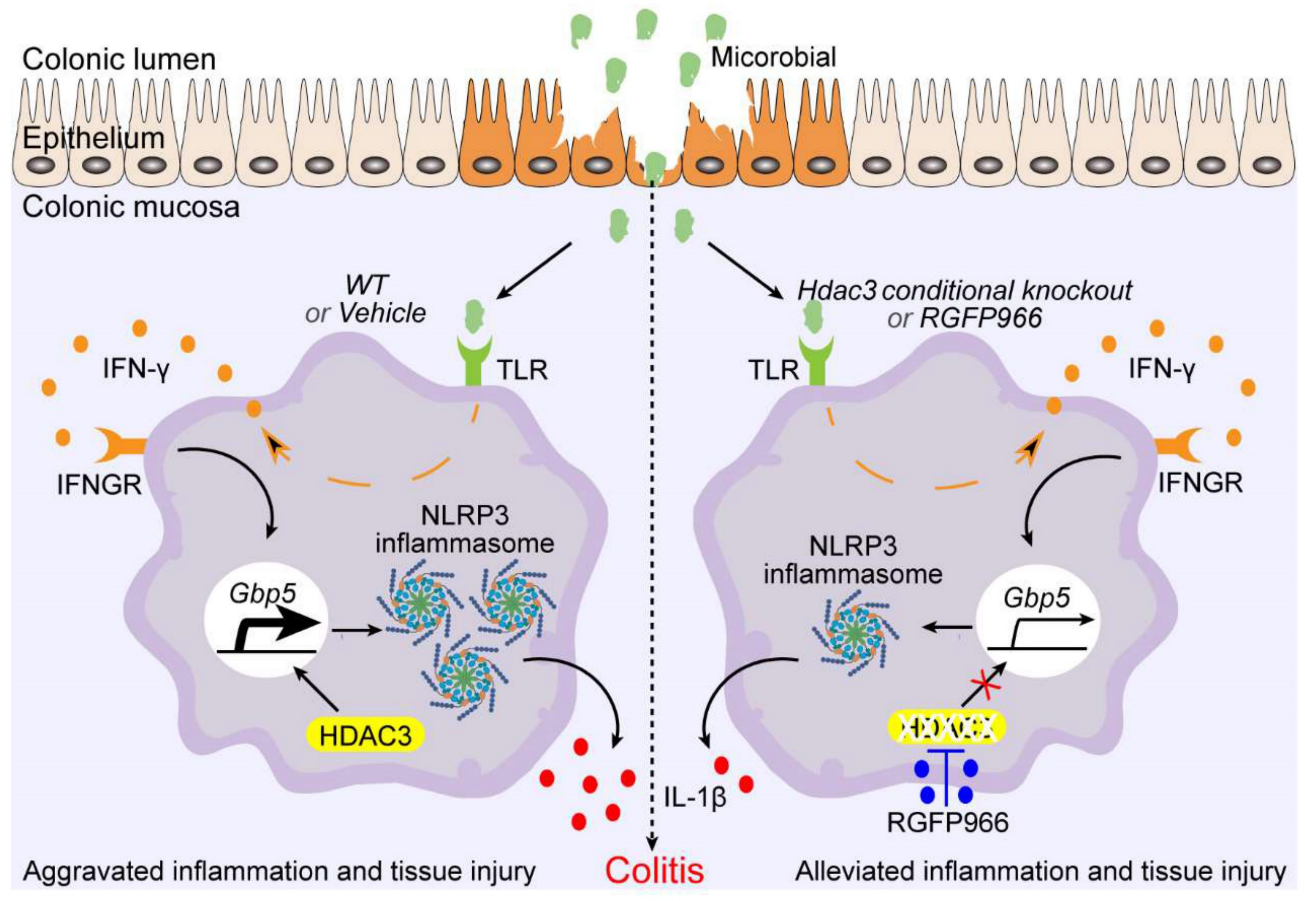
**Schematic graph of mechanism of *Hdac3* deficiency in macrophages that alleviates the disease severity of colitis.** During the development of inflammatory bowel disease (IBD), microbiota activate the expression of IFN-γ via toll-like receptors, then the IFN-γ is secreted to induce the expression of anti-infection genes. Gbp5 is one of its downstream genes that was found to be regulated by HDAC3 in our study. Conditional knockout of *Hdac3* in CX3CR1-positive monocytes (including macrophages) or inhibition of HDAC3 by RGFP966 attenuated the disease severity and inflammation of DSS-induced colitis. In addition, inhibition of HDAC3 resulted in decreased transcriptional activity of IFN-γ induced expression of GBP5, and restrained GBP5-dependent activation of NLRP3 inflammasome. In conclusion, inhibition of HDAC3 in macrophages could ameliorate the disease severity and inflammatory response in colitis by regulating GBP5-NLRP3 axis, identifying a new therapeutic avenue for the treatment of colitis.

By deacetylating the histone and non-histone proteins, HDAC3 regulates the expression and activities of plenty of genes, then plays multiple roles in different type of cells 30. In the current study, we found HDAC3 promoting IFN-γ induced expression of GBP5, which in turn, potentiated the activation of NLRP3 inflammasomes and aggravated the disease severity of DSS-induced colitis. Loss of HDAC3 in CX3CR1 positive monocytes or macrophages attenuated the activation of inflammasomes and tissue damage in mouse colitis (Figure 8). However, loss of HDAC3 in the IEC is reported to produce a more severe colonic tissue injury in colitis 1. A follow up study reveals that intestinal epithelial HDAC3 is essential for directing the dynamic balance of regulatory T cells (Tregs) to recognize commensal microbes and control inflammation 31. And loss of intestinal epithelial HDAC3 would decrease the commensal-specific Tregs, resulting in more severe disease 31. These findings suggest that HDAC3 in different cells may play distinct roles in the same disease. Interestingly, HDAC3 functions as an enhancer in immune activation in both IEC- and CX3CR1-positive monocytes. In IEC, HDAC3 promotes the activation of NF-κB pathway under the stimulation of commensal microbes. The activation of NF-κB upregulates the expression of major histocompatibility complex II (MHCII), and then MHCII helps the Tregs restrain the aberrant immune response 31. In the current study, we found HDAC3 facilitates IFN-γ induced expression of GBP5 in macrophages and microglia, which then potentiated the activation of NLRP3 inflammasome. In addition, administration of RGFP966 via intraperitoneal injection displayed ameliorated disease severity in our study, suggesting that it's feasible to target CX3CR1 positive monocytes HDAC3 for colitis therapy with little effect on IEC HDAC3 by changing the administration route of HDAC3 inhibitor. This conclusion supports a previous study, that homeodomain-interacting protein kinase 2 (HIPK2) binds and phosphorylates HDAC3 at serine 374 to inhibit its enzymatic activity and to restrain excessive inflammation, and thus may provide a new therapy for colitis 32.

Macrophages and microglia are the major types of CXCR1-positive monocytes, and microglia are macrophage-like immune cells, which are also activated in the development of DSS-induced colitis 33. In addition, a previous study has analyzed the differently expressed genes (DEGs) between *Hdac3*-deficient macrophages and WT macrophages by microarray 13. To screen the DEGs that are regulated by HDAC3 in both macrophage and microglia, we choose microglia for RNA sequencing, and compared the results with the microarray results of *Hdac3*-deficient macrophages. GBP5 is one of the genes that are consistently downregulated in the RNA sequencing results of *Hdac3*-deficient microglia and in the microarray results of *Hdac3* knockout macrophages. Previous studies reveal that the expression of GBP5 is significantly upregulated in a large amount of cell types under the stimulation of IFN-γ 34, 35. During infection, IFN-γ could induce a transcriptional memory on the promoter of GBP5 for the robust expression of GBP5 19. Then, GBP5 help immune cells detect LPS and other pathogen-associated molecular patterns by enhancing the activation of inflammasomes and caspases 18, 36, 37. However, aberrant activation of the GBP5-NLRP3 inflammasome axis is found to drive the development of colitis and phlebitis 23, 38. In addition, increasing evidence indicates that NLRP3 inflammasomes are involved in the development of IBD 39. In our study, we found that HDAC3 promotes the activation of NLRP3 by upregulating IFN-γ-induced expression of GBP5. Inhibition of HDAC3 will restrain IFN-γ-induced expression of GBP5, diminishing the further activation of NLRP3 inflammasomes *in vitro* (Figure 6H and I).

Different from HDAC3 promoting the activation of NLRP3 inflammasomes and IL-1β dependent inflammation by deacetylating long chain 3-hydroxyacyl-CoA dehydrogenase (HADHA) in a previous study 40, here, we display that the effect of HDAC3 on the activation of NLRP3 is environment-dependent. A similar environment-dependent phenomenon is also observed in cells under oxidative stress. Knockout of HDAC3 in macrophages significantly increased the activation of NLRP3 inflammasomes in cells pretreated with MPP^+^. Compared to the wild type treated with LPS, ATP plus rotenone, HDAC3 knockout cells produced more cleaved Caspase 1 and cleaved IL-1β 40. In consistent, the concentration of secreted IL-1β is also higher in the supernatant from HDAC3 knockout cells treated with LPS, ATP plus rotenone 40. These contradictions indicate that the role of HDAC3 on NLRP3 inflammasome activation is environment-dependent. In our study, we identified IFN-γ as one of the environmental factors that potentiate the activation of NLRP3 inflammasomes in HDAC3-dependent upregulation of GBP5.

## Conclusion

In summary, our study revealed that HDAC3 is upregulated in the colonic mucosa during the development of DSS-induced colitis, and that inhibition of HDAC3 with RGFP966 via intraperitoneal injection and conditional knockout of HDAC3 in CX3CR1-positive monocytes could attenuate DSS-induced tissue damage. Mechanically, HDAC3 facilitates the transcription of GBP5 under the stimulation of IFN-γ, then potentiates the activation of NLRP3 inflammasomes. Inhibition of HDAC3 results in decreased expression of GBP5 in macrophages, and dampens the activation of NLRP3 inflammasomes in IFN-γ-rich environments. Therefore, the CX3CR1-positive monocytes HDAC3 may be a therapeutic target for colitis.
